# Semaphorin3A Rewires CD4^+^ T-Cell Metabolism via AKT/mTORC1 Inhibition in Health and Rheumatoid Arthritis

**DOI:** 10.3390/ijms262211160

**Published:** 2025-11-19

**Authors:** Raeda Mubariki, Nasren Eiza, Adi D. Sabag, Shiri Keret, Doron Rimar, Gleb Slobodin, Devy Zisman, Elias Toubi, Zahava Vadasz

**Affiliations:** 1 Proteomic Unit, Bnai Zion Medical Center, Haifa 3478403, Israel; 2 Rappaport Faculty of Medicine, Technion, Haifa 3478403, Israel; 3 Rheumatology Unit, Bnai Zion Medical Center, Haifa 3478403, Israel; 4 Rheumatology Out-Patient Clinic, “Lin”-Clalit Health Services (OMG), Haifa 3478403, Israel

**Keywords:** Sema3A, activated T cells, immunometabolism, homeostasis, rheumatoid arthritis, oxidative phosphorylation, fatty acid metabolism

## Abstract

Semaphorin3A (Sema3A) is a regulatory protein found to be expressed on regulatory T and B cells and also secreted into peripheral blood. It has been identified as a potent immune regulator; however, not all its regulatory mechanisms have been evaluated. In this respect, we aim to investigate how Sema3A affects key metabolic pathways in T cells during homeostasis and rheumatoid arthritis (RA), and on the AKT/mTORC1 signaling axis. In this study, peripheral blood samples were collected from 119 healthy donors and 32 rheumatoid arthritis patients. T cells were subjected to Seahorse analysis to evaluate OXPHOS and glycolysis, live cell TMRE staining to evaluate mitochondrial activity, mass spectrometry for metabolite profiling, ATP determination to study ATP production, and Western blot analysis to investigate the signaling pathway activity. This study presents evidence showing that Sema3A inhibits the AKT/mTORC1 pathway, leading to a decreased glucose uptake and glycolysis disruption. Furthermore, we show that Sema3A reduces mitochondrial capacity and OXPHOS in activated T cells of healthy and RA donors, leading to a decreased ATP production. In contrast, Sema3A upregulates fatty acid oxidation (FA), probably as a backup pathway to ensure cell survival. Results with *p* values of <0.05 were considered significant. Our data may point to Sema3A’s ability to convert activated T cells’ metabolic profile back to its non-activated state. This may suggest that Sema3A might be a beneficial treatment for immune-mediated diseases by metabolically reprogramming activated T cells.

## 1. Introduction

Immune responses against self and non-self should be balanced in order to prevent exaggerated immune mediated inflammation [1]. When this balance is disrupted and regulatory mechanisms fail to maintain self-tolerance, autoimmunity and immune-mediated diseases may evolve [2,3].

The transitions between quiescent and activated immune responding cells require the use of nutrients in different metabolic pathways to produce ATP to support the functional changes in cells [4]. The integration of metabolism with immunity, known as immunometabolism, is a continuously increasing area of research, in which it studies the interface between immune responses and metabolism. Research over the past decade has established metabolism, both in health and disease [5,6].

During homeostasis, naïve T cells residing in the thymus rely on oxidative phosphorylation (OXPHOS) and fatty acid oxidation (FAO) in order to produce energy for their survival [3,7]. Once a mature naïve T cell leaves the thymus, it becomes dormant in the lymphoid organs and circulation until it is triggered by antigen exposure to activate, proliferate, and differentiate into effector T cell subsets. These processes require the switch between different metabolic pathways, as well as the changes in the amount and type of nutrients taken up, which is referred to as metabolic reprogramming [3,7,8,9,10].

T-cell activation is variably involved in autoimmune diseases, thus producing different pro-inflammatory cytokines. In RA, auto-reactive T cells produce mainly TNF and IL-17, whereas in SLE, TNF is much less involved. Unlike healthy effector T cells, T cells in RA have a low glycolytic flux due to the downregulation of the glycolytic enzyme PFKFB3, which has been termed the anti-Warburg effect. On the other hand, elevated levels of hexokinase-2 (HK2), another glycolytic enzyme, have been reported in lymphocytes infiltrating the joints of RA patients. As previously mentioned, during homeostasis resting CD4^+^ T cells utilize OXPHOS and FAO as a source of generating energy, while activated CD4^+^ T cells conduct aerobic glycolysis. In CD4^+^ T cells of RA, the downregulation of PFKFB3 results in low glycolytic flux, ATP, lactate, and pyruvate, therefore in turn reducing mitochondrial metabolism [11,12]. In addition, T cells can upregulate glucose-6-phosphate dehydrogenase (G6PD) which shunts glucose into the PPP, leading to the increased levels of NADPH which in turn neutralizes ROS. Although ROS are damaging at high concentrations, they are otherwise essential for T-cell activation and for suppressing inflammation. Reduced intracellular ROS levels occurs due to disfavored mitochondrial activity and hypoxia caused by the high number of infiltrating proliferative cells to the inflamed joint [3,7,12,13].

The role of immune semaphorins in regulating immune responses is widely reported, as is their ability to prevent immune-mediated inflammation and to maintain self-tolerance [14,15]. In a seminal study, our group reported Sema3A serum levels to be significantly lower in SLE and RA patients and that its reduced activity may tip the balance toward autoimmunity [16]. Furthermore, reduced serum levels of Sema3A were negatively correlated with SLE disease activity, renal involvement, and the detection of specific autoantibodies. Similarly, the expression of Sema3A on Tregs from RA patients was also reduced in association with disease severity and increased pro-inflammatory cytokines [17]. Finally, Vadasz et al. showed that when recombinant Sema3A was injected into NZB/W mice (mouse model of SLE) it had a beneficial effect in improving their survival and glomerular damage [15].

Catalano investigated the immunomodulatory role of Sema3A in RA using both a mouse collagen-induced arthritis (CIA) model and ex vivo assays with human RA T cells. They showed that Sema3A administration in mice reduced arthritis incidence and disease severity, accompanied by a decrease in IFN-γ and IL-17 serum levels and an increase in IL-10 levels. In parallel, Catalano et al. showed that CD4^+^ T cells from RA patients exhibited reduced Sema3A and elevated neuropilin-1 (NRP-1) expression. When they treated CD4^+^ T cells with Sema3A, there was an increase in IL-10 production and a decrease in CD4^+^ T-cell proliferation [18]. Together, these finding show that Sema3A modulates cytokine production and promotes regulatory T-cell functions, thereby attenuating inflammatory responses in arthritis [10]. With all this in mind, we assumed that the effect of Sema3A on the metabolism of T cells in health and autoimmunity should be assessed.

## 2. Results

### 2.1. Sema3A Downregulates OXPHOS and Mitochondrial Membrane Potential in Activated T Cells

Looking at Sema3A’s effect on different CD4^+^ subsets, Sema3A was reported by us and others to suppress Th1, Th17 proliferation, and Th2 activation, and reduce pro-inflammatory cytokine production (IFN-γ, IL-17, and IL-4) [14,17,19,20,21,22,23]. On the other hand, CD4^+^CD25^+^Foxp3^+^ Treg percentage was significantly increased, as well as higher IL-10 and TGF-β expression when cultured with Sema3A [22,24].

In 2006, a study showed that Sema3A is secreted by activated T cells but not by resting T cells, where it plays an immunomodulatory role by inhibiting T cell proliferation in DC/T cell clusters [23]. Indeed, when we purified CD4^+^ T cells from healthy donors and looked at the proliferation of stimulated and unstimulated CD4^+^ T cells that were co-cultured with Sema3A or NSPI conditioned medium (Sema3A-CM or NSPI-CM). Sema3A significantly reduced CD4^+^ T-cell proliferation as early as one day after activation (Supplemental Figure S1). Therefore, the next step was to look into whether Sema3A affects T-cell metabolism and how.

Previous studies showed that naïve T cells are mostly metabolically quiescent. Upon stimulation they increase their size and undergo metabolic reprogramming, switching to glycolysis to support growth, proliferation, and effector functions [24]. Activated T cells augment glycolysis and OXPHOS with a greater reliance on glycolysis at the relative expense of energy derived from OXPHOS [25].

Purified CD4^+^ T cells from healthy donors were divided into three groups that were either left unstimulated or activated for 24 h or 48 h for time kinetics. Using a mitochondrial stress test, we measured the OCR of each group. A representative graph showing the increased utilization of OXPHOS in activated T cells in comparison to the unstimulated cells is demonstrated in Supplemental Figure S2A. We then measured three different variables: basal respiration, which is the rate of oxygen uptake at rest; ATP production following oligomycin injection which inhibits complex V-ATP synthase; and maximal respiration following injection of the uncoupling agent FCCP. All these parameters were significantly increased upon all activation time points. Therefore, we decided that a 24 h activation duration is sufficient to detect changes in the mitochondrial respiration rate (Supplemental Figure S2B).

**Figure 1:**
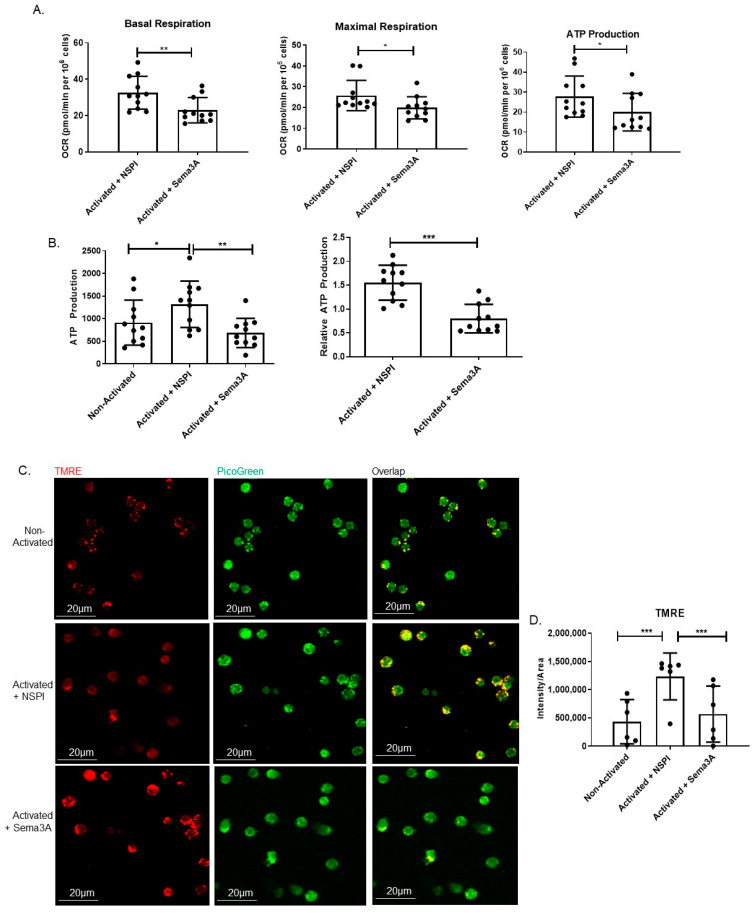
Sema3A downregulates OXPHOS and mitochondrial membrane potential. (**A**) Shows the measurement of three parameters: basal and maximal respiration as well as ATP production. Results were obtained from 11 healthy donors. (**B**) Measuring ATP production using an ATP determination kit after 12 h of activation. Results were obtained from 11 healthy donors. (**C**) Mitochondrial staining using TMRE is seen in red, and double-stranded DNA staining using PicoGreen is seen in green. Yellow is the overlap between red and green. Images were captured using a 60× oil objective lens. Scale bar = 20 µm (**D**) Statistical analysis of fluorescence intensity/area was obtained from 200 cells per group from 6 healthy donors. Mann–Whitney and Kruskal–Wallis tests. *p* value * < 0.05, ** < 0.005, *** < 0.0005.

According to the literature, Sema3A’s secretion and expression in stimulated T cells is upregulated [17,26]. Studies conducted in our lab strengthened the fact that Sema3A is a key regulatory immune player, especially in autoimmune diseases, e.g., SLE and RA patients [15,17]. These findings encouraged us to explore Sema3A’s effect on the main metabolic pathways of T cells, starting with its effect on the mitochondrial function; therefore, we conducted a mitochondrial stress test. As can be shown, Sema3A significantly decreased the basal and maximal respiration as well as ATP production when compared to the activated controls, which means that Sema3A downregulates mitochondrial respiration (Figure 1A). Further, we evaluated Sema3A’s effect on ATP production. Similarly to the above, we show again that activated T cells produce higher ATP in comparison with unstimulated T cells, which was significantly reduced when cultured with Sema3A. Results are represented as absolute and relative to the unstimulated cell group (Figure 1B).

Augmented OXPHOS activation in stimulated T cells in comparison with unstimulated T cells and its decreased activity upon Sema3A treatment suggest the possibility for coordinate changes in mitochondrial dynamics. To evaluate mitochondria in living T cells, three groups of T cells were simultaneously stained with TMRE and PicoGreen, a double-stranded DNA intercalation dye that marks double-stranded DNA. In agreement with increments in OCR in activated T cells, we show increased TMRE fluorescence intensity (Figure 1C). These results reflect an increase in proton gradients within the mitochondria. Statistical analysis shows a significant increase in the fluorescence intensity/area of activated vs. unstimulated T cells (Figure 1D). As can be shown, following Sema3A treatment, TMRE staining is significantly decreased. This result is in line with our previous results which showed that T-cell stimulation leads to an increase in the mitochondrial membrane potential and OXPHOS, and that Sema3A significantly decreases both parameters in activated T cells.

Another important aspect that can be used as an indicator of the cell’s ability to utilize aerobic glycolysis is known as the glycolytic reserve (GR). GR is a measure of the ability of activated T cells to convert pyruvate to lactate in order to meet their ATP demands through aerobic glycolysis. It also indicates that glucose-derived pyruvate is entering mitochondria and being used to fuel the TCA and support mitochondrial ATP synthesis. When comparing between activated and unstimulated T cells, we showed that activated cells have significantly higher GR, whereas Sema3A-treated cells demonstrate significantly decreased GR in comparison to the control counterparts, indicating that Sema3A not only negatively affects OXPHOS but also downregulates ATP production and glycolysis in activated T cells (Supplemental Figure S3).

### 2.2. Sema3A Downregulates Glycolysis in Activated T Cells

**Figure 2:**
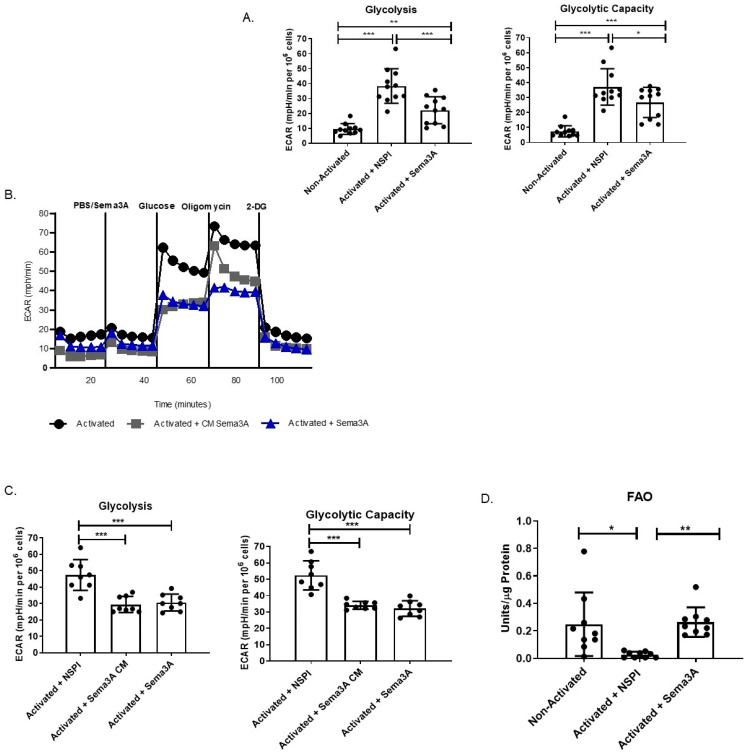
Sema3A downregulates glycolysis and increases FAO in activated T cells. (**A**) Shows the measurement of glycolysis and glycolytic capacity. Results were obtained from 11 healthy controls. (**B**) Demonstrates a representative ECAR graph of activated T cells treated with NSPI-CM, Sema3A-CM, or acute injection. (**C**) Shows the measurement of glycolysis and glycolytic capacity. Results were obtained from 8 healthy control. (**D**) Fatty acid oxidation assay shows the FAO of unstimulated and activated T cells cultured with or without Sema3A. Results were obtained from 9 healthy donors. Kruskal–Wallis test. *p* value * < 0.05, ** < 0.005, *** < 0.0005.

Activated T cells rely mainly on glycolysis to produce the needed energy for cell survival and function. We anticipate that similar to its effect on OXPHOS, Sema3A downregulates glycolysis in activated T cells as well. To test this theory, we evaluated Sema3A’s effect on glycolysis using the glycolysis stress test. Two main parameters were measured, the overall glycolysis and the glycolytic capacity—which is a measure of the maximum conversion rate of glucose to pyruvate or lactate and the maximum capacity of ATP generation. We show that our results are similar to those in the literature, meaning activated T cells have a significantly higher glycolytic performance and have higher capacity to convert glucose into pyruvate in order to produce energy. Here, we demonstrate that Sema3A’s addition significantly reduced the cell’s ability to perform glycolysis in comparison with the control (Figure 2A). To prove that the downregulation of glycolysis is indeed a result of Sema3A’s function, we purified the Sema3A protein and acutely injected the cells with either the purified protein or with PBS. We show that the purified protein has the same effect that was shown when using CM containing Sema3A, proving that the downregulation of the metabolic pathways is indeed due to the Sema3A protein (Figure 2B,C). These results show that Sema3A significantly downregulates two main metabolic pathways, namely, OXPHOS and glycolysis, leading to decreases in ATP production in activated T cells.

### 2.3. Immmunometabolic Reprogramming: Sema3A Increases FAO

Since the results so far prove that Sema3A downregulates glycolysis and OXPHOS, we were interested in investigating how activated T cells produce the necessary ATP. We hypothesized that there is an alternative pathway that activated T cells switch to in order to ensure their survival. We mentioned earlier that unstimulated cells rely on OXPHOS, TCA, and FAO [3,7]. We conducted a FAO assay. Similarly to the literature, we show that unstimulated T cells have significantly higher FAO than activated T cells. Interestingly, we show that Sema3A upregulates FAO in activated T cells (Figure 2D).

Thus, Sema3A may appear to convert the metabolic profile of activated T cells back to the unstimulated cell state. This assay is based on the oxidation of octanoyl-CoA (a medium-chain fatty acid), which is coupled with NADH-dependent reduction and formazan formation [27]. Future studies should be conducted to evaluate the full effect of Sema3A on FAO.

### 2.4. Sema3A Downregulates Key Metabolites During Glycolysis

**Figure 3:**
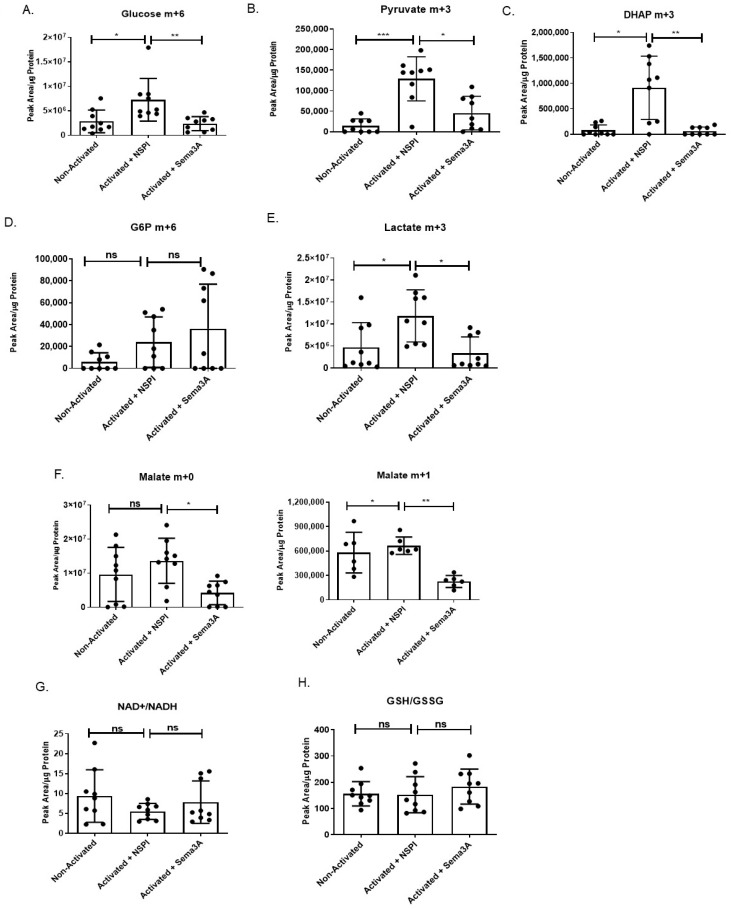
Sema3A inhibits glucose uptake and glycolysis but does not induce oxidative stress. (**A**–**F**) Glycolysis and TCA cycle metabolites and intermediates. (**G**,**H**) NAD^+^/NADH and GSH/GSSG ratios. Results were obtained from 9 healthy donors from 3 independent repetitions. Kruskal–Wallis test was used. *p* value * < 0.05, ** < 0.005, *** < 0.005, ns—not significant.

For in-depth insights on Sema3A’s activity and its potential metabolic targets, unstimulated and activated T cells treated with NSPI-CM or Sema3A-CM were subjected to metabolic scan using LC/MS. Changes in primary metabolites that play a key role in glycolysis and TCA were measured. Analyzing glycolysis, we show that there is a significant increase in the glucose uptake upon T cells’ activation in general. However, Sema3A-treated cells demonstrated a significant reduction in glucose uptake into the activated cell. Interestingly, glucose-6-phosphate (G6P) was not affected by Sema3A, in contrast to the reduction of dihydroxyacetone phosphate (DHAP), which is an important intermediate in both glycolysis and lipid biosynthesis. The overall outcome of the glycolytic pathway disturbance upon Sema3A treatment was a significant reduction in pyruvate and lactate production (Figure 3A–E). To validate the fact that Sema3A decreases glucose uptake, we conducted a glucose consumption assay. Cell media was collected and remnant glucose levels were evaluated. The remnant glucose level in the media of Sema3A-treated cells is significantly higher than the control counterparts (Supplemental Figure S4A). In addition, the cell media collected from the glucose tracing experiment were analyzed. Looking at the labeled remnant glucose levels in the cell media, we show that there is a significant decrease in the remnant glucose of activated T cells that were not treated with Sema3A compared with the treated cells. On the same note, when we calculated the amount of glucose uptake (media without cells minus media with cells) we again showed a significant increase in glucose uptake in activated T cells and a significant decrease in Sema3A-treated cells (Supplemental Figure S4B). These results further prove that Sema3A inhibits glucose consumption in activated T cells.

Unlike the clearly noticeable effect of Sema3A on the glycolytic pathway, we observed little to no changes in the TCA. Citrate levels were increased upon T-cell activation in comparison to the unstimulated counterparts without any effect of Sema3A (Supplemental Figure S5).

Although the fractional labeling of the TCA metabolites from glucose was not detected, the overall TCA metabolite pool sizes were preserved. Interestingly, we show that Sema3A negatively affects malate levels, which is important for NADH production (Figure 3F). It is worth noting that Sema3A’s effect on some essential and non-essential amino acids were also investigated, but no significant changes were detected). Taken together, our results indicate that Sema3A negatively affects glucose uptake, glycolysis, and the last part of the TCA cycle, but does not affect amino acid metabolism.

Due to our study findings, which show that Sema3A suppresses both glycolysis and mitochondrial respiration while increasing FAO, it is crucial to investigate whether Sema3A induces oxidative stress due to ROS increase in cells. Thus, we looked at two indicators of oxidative stress, one being the NAD^+^/NADH ratio, in which the consequence of imbalance leads to oxidative stress and oxidative damage [28], and the other being the GSH/GSSG ratio, which is a potential analytical tool that reflects disturbances in redox metabolism [29]. Our results show that there are no significant changes in the NAD^+^/NADH and GSH/GSSG ratios between the three different groups (Figure 3G,H), indicating that redox homeostasis remains stable and that while Sema3A suppresses metabolic pathways it does so without inducing oxidative stress.

### 2.5. Sema3A’s Effect on the PI3K/AKT/mTORC1 Signaling Pathway

**Figure 4:**
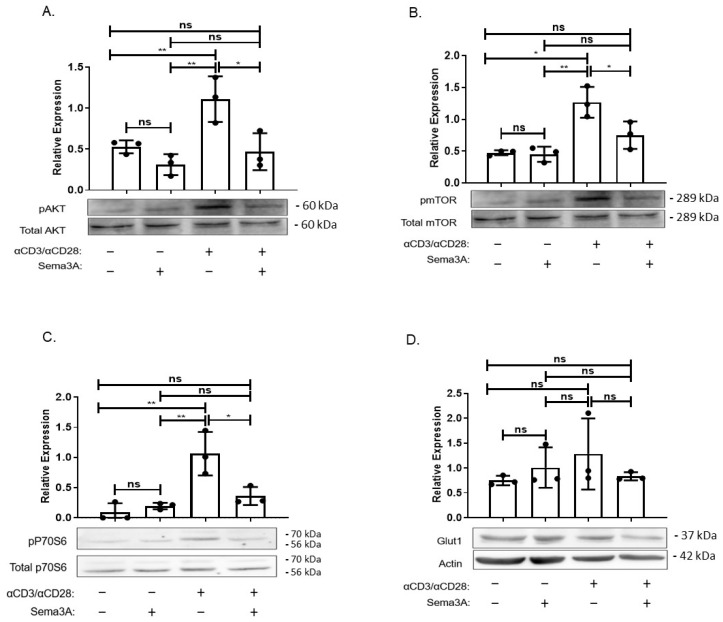
Sema3A inhibits AKT/mTORC1 signaling pathway. (**A**–**D**) Sema3A’s effect on the intracellular signaling pathway was assessed by immunoblotting for the indicated phosphoproteins in unstimulated and 24 h-activated T cells treated with EB or purified Sema3A protein. Results were obtained from 3 independent experiments. Kruskal–Wallis. *p* value * < 0.01, ** < 0.001, ns—not significant.

The major question of how do cells sense and adapt to the nutrients available in their environment remains somewhat incompletely understood. What is known is that one key pathway is the signaling pathway anchored by mTORC1 kinase [30]. Since our results show that Sema3A downregulated glucose consumption and negatively affected DHAP levels in activated T cells, we investigated Sema3A’s effect on the intracellular signaling pathway of PI3K/AKT/mTORC1 and whether it directly affects mTORC1 activity. We used specific antibodies directed to the phosphorylated forms of mTORC1, AKT, and ribosomal S6 kinase (pS6K), a downstream target of mTORC1 that plays a key role in regulating cell growth by controlling the translation and biosynthesis of proteins [31]. Purified T cells from healthy donors were divided into four groups, two unstimulated groups and two activated groups, and each subgroup was treated either with purified Sema3A or elution buffer (EB). Our results showed that activated T cells upregulate the phosphorylation of AKT, mTORC1, and its downstream target pS6K in comparison with unstimulated T cells. However, Sema3A significantly downregulated the phosphorylation of all three molecules of the signaling pathway, indicating that Sema3A does not affect mTORC1 solely but rather has a more upstream effect. The most surprising result was that Sema3A reduces the expression levels of Glut-1; however, the reduction was not statistically significant (Figure 4A–D).

In this respect, it would indeed be important to perform receptor rescue or inhibition experiments to further strengthen our findings. Although such experiments were not conducted in the present study, this issue has been addressed in several independent reports. Stöckl et al. demonstrated that stimulation of human osteoarthritic chondrocytes with recombinant Sema3A led to a rapid reduction in AKT phosphorylation. This effect was abolished when neuropilin-1 (NRP1), the canonical Sema3A receptor, was knocked down, thereby showing that Sema3A signals via NRP1 to suppress PI3K/AKT pathway activation in these cells [32]. Similarly, Sumi et al. observed that in ATDC5 chondrocytes exposed to high-magnitude cyclic tensile strain (CTS) to induce an inflammatory response, subsequent treatment with recombinant Sema3A suppressed pAKT expression and reduced inflammatory gene expression, leading to decreased cytokine production [33]. These are similar to the results obtained from previous studies conducted by our team. Furthermore, Sang et al. reported that culturing podocytes with recombinant Sema3A reduced AKT phosphorylation and downstream survival signaling, resulting in increased apoptosis [34]. Thus, based on these findings we believe that the inhibitory effect on the signaling pathway is due to Sema3A’s activity. Together, these studies provide strong evidence that Sema3A can modulate AKT/mTORC1 signaling through NRP1 in multiple cell types. Based on these findings and the similarity of our results, we infer that the inhibitory effect observed in our current study is due to Sema3A’s receptor-mediated activity. Future experiments will be necessary to confirm that the observed effects are specifically mediated through the AKT/mTORC1 signaling axis.

### 2.6. Sema3A Inhibits OXPHOS in RA Patients

**Figure 5:**
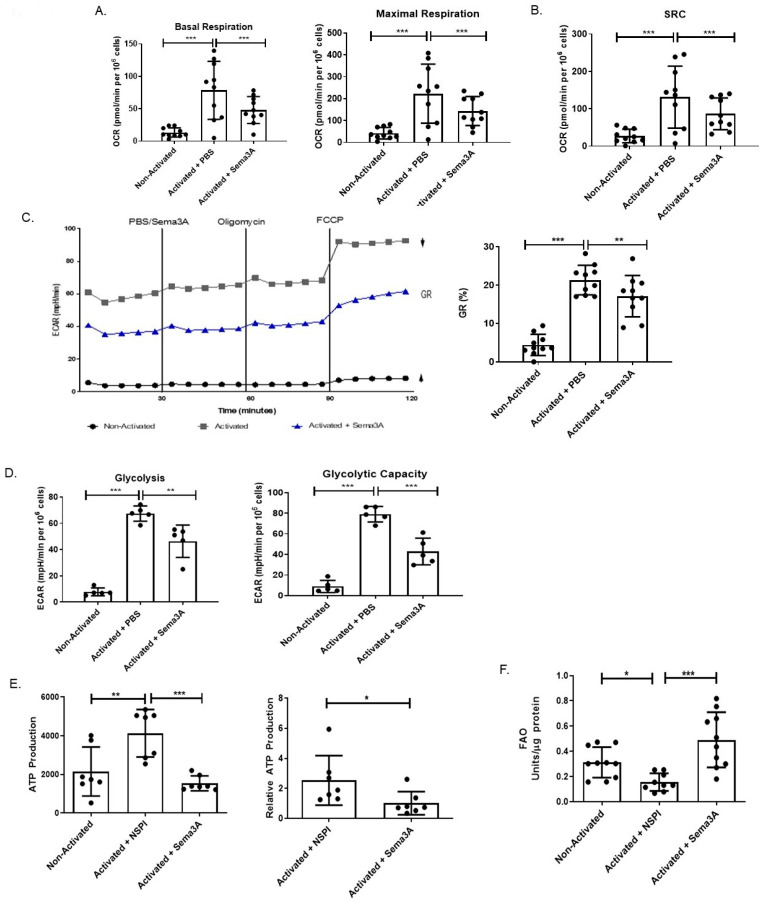
Sema3A inhibits OXPHOS, glycolysis, and ATP production while increasing FAO in CD4^+^ T cells of RA patients. (**A**) Shows the measurement of the basal and maximal respiration. (**B**) Shows the spare respiratory capacity. (**C**) ECAR at baseline and after sequential treatment with oligomycin and FCCP. GR, glycolytic reserve. Results were obtained from 10 RA patients. (**D**) Shows the measurement of the glycolysis and glycolytic capacity. Results were obtained from 5 RA patients. (**E**) The graph shows the absolute and relative ATP production of unstimulated and stimulated T cells after a 12 h activation period with or without Sema3A. Results were obtained from 7 RA patients. (**F**) Fatty acid oxidation assay shows the FAO of unstimulated and activated T cells cultured with or without Sema3A. Results were obtained from 10 RA patients. Mann–Whitney and Kruskal–Wallis tests. *p* value * < 0.05, ** < 0.005, *** < 0.0005.

Studies showed that abnormal metabolic pathways are involved in RA. Therefore, the purpose of this part of the research was to evaluate the metabolic changes in activated T cells taken from RA patients as a prototypic immune disease. Specifically, the aim was to evaluate Sema3A’s metabolic effect on these cells. Starting with OXPHOS, purified T cells from RA patients were divided into three groups, unstimulated and activated T cells either treated with Sema3A or PBS. Our results show that similar to healthy donors, activated T cells from RA patients increase the basal and maximal respiration in comparison to the unstimulated group and that Sema3A leads to the significant decrease in both parameters (Figure 5A).

Another important parameter to measure is called “spare respiratory capacity” (SRC). SRC characterizes the extra mitochondrial capacity available in a cell to produce energy in response to acute cellular stress or increased workload, thereby avoiding an ATP crisis [35,36]. When measured, we showed that upon activation there was a significant increase in the SRC when compared with unstimulated T cells. Such an increase was not detected in activated T cells that were treated with Sema3A (Figure 5B). Looking at the GR and comparing between activated and unstimulated T cells, we show that activated cells have significantly higher GR, whereas Sema3A-treated cells demonstrate significantly decreased GR in comparison to the control counterparts (Figure 5C). Taken together, these results prove that Sema3A not only negatively affects OXPHOS but also negatively affects the cell’s ability to produce energy if the need arises, and that it may also inhibit glycolysis in activated T cells taken from RA patients.

### 2.7. Sema3A Downregulates Glycolysis in RA Patients

Unlike healthy effector T cells, T cells in RA have a low glycolytic flux due to the downregulation of the glycolytic enzyme PFKFB3, which has been termed the anti-Warburg effect [11,12]. Therefore, our aim was to understand if Sema3A affects glycolysis in RA as well. In a glycolytic stress test we obtained similar results to those we showed in healthy donor T cells: we observed close to no glycolytic activity in unstimulated T cells when comparing them to activated T cells. Furthermore, Sema3A treatment significantly reduced glycolysis in RA, as was found in healthy donors (Figure 5D).

### 2.8. Sema3A Decreases ATP Production in RA

We demonstrated that Sema3A downregulates ATP production in activated T cells of healthy individuals. When conducting an ATP determination test replicating the same working conditions in CD4^+^ T cells from RA patients, we showed that, similar to healthy donors, T cells from RA patients significantly increase ATP production upon activation. Moreover, Sema3A significantly decreases ATP production in these activated T cells. Absolute and relative results are presented in Figure 5E.

### 2.9. Sema3A Increases FAO in RA

We were interested in evaluating whether Sema3A leads to the metabolic reprogramming of RA T cells, meaning whether Sema3A upregulates FAO as an alternative pathway to ensure cell survival. To that end, we conducted a FAO assay. We show that unstimulated T cells have significantly higher FAO than activated T cells, and that activated T cells upregulate FAO upon treatment with Sema3A in comparison with the activated counterparts (Figure 5F).

## 3. Discussion

Sema3A is expressed on many immune cells including T and B cells, thus inducing their regulation by enhancing the production of inhibitory cytokines, such as IL-10, IL-35, and TGF-β. With respect to T-cell activation and differentiation, Sema3A was shown to inhibit T-cell proliferation and to reduce the production of pro-inflammatory cytokines in immune-mediated diseases such as RA and colitis. Since it is known that cell proliferation requires nutrients, energy, and biosynthetic activity, it is therefore not surprising that metabolic activities in proliferating cells are fundamentally different from those in non-proliferating cells [37].

In the current study, we investigated Sema3A’s effect on two main metabolic pathways, namely OXPHOS and aerobic glycolysis in CD4^+^ T cells. We showed that upon activation, T cells increase the utilization of the OXPHOS pathway in comparison with unstimulated T cells to meet the cells’ need for energy production, and also how Sema3A significantly inhibits OXPHOS in activated T cells, thus downregulating their activation.

ATP is the main source of energy, and the mitochondria are responsible for providing the majority of ATP involving the OXPHOS process [38]. The OXPHOS system consists of five multimeric complexes embedded in the mitochondrial inner membrane that transfer positively charged protons, resulting in a net internal negative charge known as the mitochondrial transmembrane potential. The net negative charge across a healthy mitochondrion can be detected by staining cells with positively charged dyes [39]. Using this fact, we stained T cells with TMRE and showed that activated T cells have higher membrane potential than unstimulated T cells, and that Sema3A treatment significantly reduces mitochondrial potential.

We also observed a significant decrease in the GR of activated T cells treated with Sema3A. GR defines the cell’s ability to convert pyruvate to lactate to meet their energy demands through glycolysis. Indeed, through investigating Sema3A’s effect on the glycolytic pathway we showed that Sema3A significantly decreases glycolysis in activated T cells. Using metabolite screening mass spectrometry to further understand Sema3A’s effect on OXPHOS and glycolysis, we showed that Sema3A negatively affects glucose uptake and its breakdown into pyruvate and lactate.

Interestingly, Sema3A shows no such effect on the TCA, where all metabolites remain unaffected; it does, however, effect malate. This finding is important because it is known that malate metabolism produces NADH. Electrons from NADH are then transferred to molecular oxygen to produce ATP via OXPHOS [40].

The above-mentioned results lead to the question of how these cells still manage to survive after Sema3A treatment. According to the literature, naïve T cells in quiescence are fueled by OXPHOS and FAO [7]. Since activated T cells treated with Sema3A appeared to have a metabolic profile similar to naïve T cells, we anticipated that Sema3A-treated cells also rely on FAO to ensure survival. Indeed, we showed that similar to unstimulated T cells, activated T cells when treated with Sema3A increase FAO utility, leading to the assumption that Sema3A-treated cells upregulate FAO: possibly as an alternative pathway to ensure cell survival. The metabolic shift from glycolysis toward lipid utilization is common in quiescent or memory T cells. However, we note that ATP is still downregulated, indicating that while FAO is upregulated it might function as a partial compensatory pathway since it is not enough to sustain T-cell proliferation or fully restore energy production. Therefore, further studies should be conducted in order to establish whether FAO is increased as a backup mechanism to override Sema3A’s reduction in glycolysis and OXPHOS.

As previously mentioned, mTORC1 is a sensory complex that regulates cell growth and metabolism in response to nutrients such as amino acids and glucose. The results of a recently conducted study supported the existing notion that mTORC1 can sense glucose through at least two pathways: one AMPK dependent and the other not dependent on AMPK. In the study, they found that DHAP, a metabolite which up until then was not connected to mTORC1, played a key role in its activation. They showed that DHAP is sufficient to activate mTORC1 even in the absence of glucose and that a decreased level of DHAP leads to a decrease in mTORC1 activation [31]. Since our results show that Sema3A downregulated glucose consumption and DHAP levels in activated T cells, we looked into Sema3A’s effect on the signaling pathway. In our study, we show that Sema3A negatively affects the AKT/mTORC1 activity by inhibiting the phosphorylation of the signaling molecules involved; furthermore, we also show that Sema3A inhibits the phosphorylation of P70S6, a ribosomal protein which is crucial for synthesis induction of key components that are important for cell survival and cell growth [41]. In immune cells there are three different receptors for Sema3A: Plexin A, NRP1, and CD72. Stöckl et al. demonstrated that Sema3A inhibits AKT phosphorylation via NRP1. However, it has not yet been established whether Sema3A conserves this inhibition mechanism (via NRP1 or the other receptors) in T cells. Thus, in a future study we plan to find out through which receptor Sema3A inhibits the AKT/mTORC1 signaling pathway, and if it is through direct receptor-blocking or knockdown approaches.

In respect with the above, we looked into the role of Sema3A in the metabolism of T cells derived from RA patients. Thus, we investigated OXPHOS and aerobic glycolysis and showed that unstimulated T cells have low glycolytic flux, which is significantly increased in activated cells, and that Sema3A significantly reduces glycolysis and OXPHOS in activated T cells of RA patients. We also show that Sema3A inhibits ATP production in RA activated T cells. In addition, Sema3A upregulates the FAO pathway in activated T cells of RA patients, which is similar to healthy donors.

To summarize, our results indicate that Sema3A inhibits OXPHOS, glycolysis, and ATP production in CD4^+^ T cells derived from peripheral blood of both healthy and RA patients. On the other hand, Sema3A upregulates FAO in activated T cells. Furthermore, Sema3A negatively affects mitochondrial capacity in activated T cells, downregulates glucose consumption, and inhibits the AKT/mTORC1 signaling pathway which is important for cell growth.

Importantly, NAD^+^/NADH and GSH/GSSG ratios remained unchanged across all groups, suggesting that Sema3A preserves redox homeostasis and does not induce oxidative stress.

Thus, we conclude that Sema3A may appear to convert the metabolic profile of activated T cells back to the non-activated state. This may suggest that Sema3A is beneficial in treating immune-mediated diseases by remodeling the metabolism of activated T cells.

Limitation of this study: Since we investigated the overall effects of Sema3A on major metabolic pathways, our findings open up new aspects for further, deeper investigations. Furthermore, the sample size of RA patients is relatively small because only few patients meet the criteria for inclusion; however, the results were significant. Thus, we plan to further expand this issue on more RA patients and also on other autoimmune diseases. In addition, the number of purified CD4^+^ T cells from RA patients are very low, which limits the ability to perform large experiments; therefore, we only conducted key experiments which we deemed to be most relevant to our study.

## 4. Materials and Methods

### 4.1. Preparation and Purification of Sema3A

Conditioned medium of HEK293 cells producing recombinant Sema3A fused with 6×Histidine tag in the N-terminal, or NSPI (empty vector used as a control) whose construction was previously described [18], were collected and purified using Ni-NTA agarose beads (20-30210, Qiagen, Hilden, Germany). The protein activity was assessed using the cytoskeleton collapse assay [18].

### 4.2. CD4^+^ T Cell Isolation

For the isolation of CD4^+^ T cells, 40 mL of peripheral blood samples collected from either healthy donors or RA patients were drawn to heparin-washed tubes, then loaded on Lymphoprep (07861, StemCell Technologies, Vancouver, BC, Canada) and centrifuged at 800× *g* for 30 min. The cells were washed and later CD4^+^ T cells were separated using anti-human MACS CD4 microbeads (130-045-101, Miltenyi Biotec, Bergisch Gladbach, Germany), as per the manufacturer’s instructions.

### 4.3. Extracellular Flux Analysis

For CD4^+^ T-cell activation, anti-CD3 and anti-CD28 antibodies were used (16-0038-85 and 16-0289-85, respectively, Thermo Fisher Scientific, Inc., Waltham, MA, USA). For the mitochondrial stress test, 350,000 cells were seeded in a 96 well-plate (102905-100, Agilent, Technologies Inc., Santa Clara, CA, USA) pre-coated with Cell-Tak as per the manufacturer’s instructions (FAL354240, Corning Incorporated, Corning, NY, USA), using DMEM basic media enriched with 10 mM glucose, 1 mM sodium pyruvate, and 2 mM glutamine (pH = 7.4) as described in other published metabolomics studies. Sensor cartridge ports were loaded with 2 µM oligomycin, 2.5 µM FCCP, and 2 µM Rot/AA. For the glycolysis stress test, the same procedure described above was conducted, cells, however, were cultured using glucose-free DMEM basic media supplied with 2 mM glutamine (pH = 7.4) and incubated for 2 h at 37 °C. A total of 2 µg of Sema3A was added to port A, and ports B–D were loaded with 10 mM glucose, 2 µM oligomycin, and 50 mM 2-DG, respectively. The in live oxygen consumption rate (OCR) and extracellular acidification rate (ECAR) measurements were performed using Agilent Seahorse XF analyzers, Agilent Technologies Inc., Santa Clara, California, USA. Results were normalized to cell number (post-assay).

### 4.4. TMRE Staining

Purified CD4^+^ T cells were divided into 3 groups each containing 1 × 10^6^ cells; the first group was unstimulated while the two other groups were activated using anti-CD3 and anti-CD28 and were either co-cultured with NSPI-CM or Sema3A-CM (concentration of 2 µg/mL) for 24 h at 37 °C. On the day of the experiment, the mitochondria were stained using 20 nM of tetramethylrhodamine, ethyl ester, perchlorate (TMRE) solution, (T669, Thermo Fisher Scientific Inc., Waltham, MA, USA) and the nuclei were counter stained with 3 µL/mL of PicoGreen solution using Quant-iT™ PicoGreen™ dsDNA assay kits (P7589, hermo Fisher Scientific Inc., Waltham, MA, USA). Cells were incubated with both solutions for 15 min at 37 °C. From each group, 1 × 10^5^ cells were transferred to a glass-bottom culture dish (627870,Greiner Bio-One International GmbH, Frickenhausen, Germany). Cell images were captured using a Zeiss inverted spinning disk confocal microscope, Carl Zeiss AG, Oberkochen, Germany. Image analysis was performed with FIJI (ImageJ version 1.54f; open-source distribution, University of Wisconsin–Madison, Madison, WI, USA; https://fiji.sc/, downloaded on [15 July 2024]).

### 4.5. Mass Spectrometry Analysis

Purified CD4^+^ T cells were activated with anti-CD3 and anti-CD28 cultured with either NSPI-CM or Sema3A-CM for 21 h. Cells were then washed with PBS, re-cultured with fresh NSPI-CM or Sema3A-CM containing 1 mM [^13^C] glucose (added to CM containing 5 mM glucose), and re-activated for 3 h. Metabolites were extracted using ice-cold metabolite extraction mix (methanol–acetonitrile–water (*v*/*v*/*v*, 5:3:2)). Results were normalized to total protein content.

### 4.6. Western Blot Analysis

Purified CD4^+^ T cells were cultured in regular RPMI with or without activators for 24 h in 37 °C. Cells were then incubated and rotated either with purified 2 µg/mL Sema3A or elution buffer for 15 min on ice, then for 5 min in 37 °C. Cells were then harvested and lysed using phosphorylation lysis buffer (150 mM NaCl, 1 mM EDTA, 50 mM Tris-Cl pH 7.5, 1% Triton, and 0.4% Sodium deoxycholate) with protease (P8340, Sigma-Aldrich Chemie GmbH, Taufkirchen, Germany) and phosphatase inhibitor cocktails (P2850, Sigma-Aldrich Chemie GmbH, Taufkirchen, Germany). Equal amounts of proteins (100 µg) from samples were subjected to 10% SDS-PAGE and immunoblotted with an antibody directed against a total and phosphorylated target protein. Actin was used as a loading control to confirm equal protein input. The bound antibodies were visualized using the EZ-ECL method (20-500-120, Sartorius AG, Göttingen, Germany); next the blots were viewed by ImageQuant Reader LAS4000, Version 7.0.16, Cytiva/GE Healthcare Life Sciences, MA, USA and the quantification of band intensity was performed using ImageQuant TL analysis software, Version 10.2, Cytiva/GE Healthcare Life Sciences, MA, USA.

### 4.7. ATP Determination Assay

Purified CD4^+^ T cells were divided into 3 groups, as previously described, and cultured only for 12 h at 37 °C. Cells were then washed twice with PBS. The cell pellets were re-suspended using 100 µL PBS and boiled at 95 °C for 5 min. Cells were then centrifuged at 13,000 rpm for 10 min. Supernatants were collected and processed in an ATP determination kit (A22066, ThermoFisher Scientific Inc., Waltham, MA, USA), as per the manufacturer’s instructions. The results were normalized by the ATP production in unstimulated cells.

### 4.8. Fatty Acid Oxidation (FAO)

Purified CD4^+^ T cells were divided into 3 groups, as previously described. On the day of the experiment, cells were washed twice with ice-cold dwH_2_O, lysed, and plated as per the manufacturer’s instructions (BR00001, AssayGenie, Ireland Ltd., 25 Windsor Place, Dublin 2, County Dublin, Ireland). Results were obtained and analyzed after a 90 min incubation in 37 °C. This assay was used in several published studies and was deemed acceptable to reflect fatty acid oxidation activity [19,20].

### 4.9. Study Population

Our study consisted of 32 RA patients who were evaluated by rheumatologists at BnaiZion Medical Center, Haifa, or Lin Outpatient Clinical Center, Haifa, according to the ACR criteria for clinical disease activity index (CDAI).

Blood samples were drawn when the patients were on mild therapy, as listed in the table, or were still not treated: all were before heavier therapies were initiated. Diabetic patients were excluded from the study (Table 1).

The healthy control group (N = 119) consisted of age- and sex-matched healthy individuals without any evidence of systemic inflammatory disease or diabetes. In this study, sex was not considered as a biological variable.

### 4.10. Statistical Analysis

For comparison of only two groups, data were analyzed using Mann–Whitney test; for more than two groups, the Kruskal–Wallis test was used.

Data displayed in dot plots are represented as means ± SEM, with individual data points from biological replicates. Statistical tests applied to the data are listed in the figure legends and were calculated using GraphPad Prism, version 8.0.2. A *p* value of less than 0.05 was considered significant.

### 4.11. Antibodies

Phospho-AKT (Ser473) antibody (#4060, Cell Signaling Technology, Danvers, MA, USA).

AKT 1/2/3 (H-136) antibody (#H2714, Santa Cruz Biotechnology, Dallas, TX, USA).

Phospho-mTOR (Ser2448) antibody (#5536, Cell Signaling Technology, Danvers, MA, USA).

mTOR antibody (#2983, Cell Signaling Technology, Danvers, MA, USA).

Phospho-p70S6 Kinase (Thr389) antibody (#9205, Cell Signaling Technology, Danvers, MA, USA).

P70S6 antibody (#9202, Cell Signaling Technology, Danvers, MA, USA).

Glut1 antibody (ab115730, Abcam, Cambridge, UK).

### 4.12. Supplemental Methods

#### 4.12.1. Cell Proliferation Assay

Purified CD4^+^ T cells either were left unstimulated or activated and treated with Sema3A-CM or NSPI-CM, then incubated for 0, 24, or 48 h at 37 °C (1 × 10^6^ per treatment). On each day of the experiment, a ready-to-use WST-1 reagent (5015944001, Sigma-Aldrich, St. Louis, MI, USA) was diluted to a 1:10 working concentration, incubated for 2 h at 37 °C, and the O.D was measured at 440 nm, as per the manufacturer’s instructions.

#### 4.12.2. Glucose Quantification Assay

Cell media from activated CD4+ T cells cultured with NSPI-CM or Sema3A-CM for 24 h were collected, snap-frozen in liquid nitrogen, and stored at −80 °C prior to assay. On the day of the experiment, the media were thawed on ice and proceeded to be processed using a glucose assay kit (ab65333, Abcam, Cambridge, UK) as per the manufacturer’s instructions. The remnant glucose amount in the media was normalized to snap-frozen conditioned medium without cells.

## Figures and Tables

**Table 1:** Characteristics of 32 RA patients.

Patients Characteristics	*n* = 32
Age (yr.)	26–78 (59)
Sex	
Males	20/32 (62.5%)
Females	12/32 (37.5%)
Disease Duration (yr.)	1–23 (8.6)
CDAI Range	19–84 (33.2)
Comorbidities	
Dyslipidemia	1/32 (3.1%)
Asthma	1/32 (3.1%)
Hypertension	5/32 (15.6%)
Hypothyroidism	2/32 (6.2%)
Ischemic Heart Disease	1/32 (3.1%)
HBV	1/32 (3.1%)
Treatments	
Tocilizumab	2/32 (6.2%)
Methotrexate	2/32 (6.2%)
Rituximab	1/32 (3.1%)
Leflunomide	1/32 (3.1%)
Prednisone	3/32 (9.4%)
Hydroxychloroquine	1/32 (3.1%)
NSAIDs	1/32 (3.1%)
Infliximab	1/32 (3.1%)

yr: years; CDAI: clinical disease activity index; HBV: hepatitis B virus; NSAIDs: non-steroidal anti-inflammatory drugs.

## Data Availability

The raw data supporting the conclusions of this article will be made available by the authors on request.

## Supplementary Materials

The supporting information can be downloaded at: https://www.mdpi.com/article/10.3390/ijms262211160/s1.
