# Supplementary material for: Semaphorin3A Rewires CD4+ T-Cell Metabolism via AKT/mTORC1 Inhibition in Health and Rheumatoid Arthritis
Source: Int J Mol Sci. 2025 Nov 19;26(22):11160. doi: 10.3390/ijms262211160 (PMC12652675; doi:10.3390/ijms262211160)
Supplement: Supplementary file 1 [file ijms-26-11160-s001.zip › ijms-3933840-supplementary.pdf]

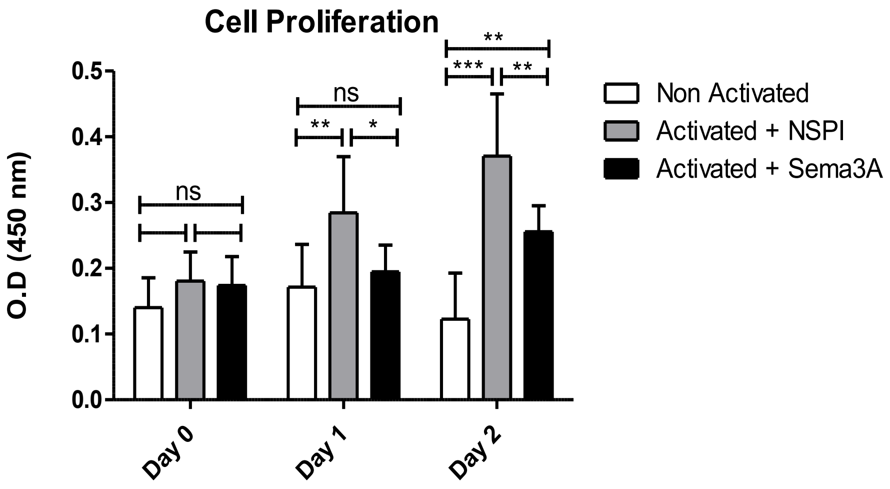

Supplemental Figure S1: Sema3A inhibits T cell proliferation. T cell proliferation at day 0, 1 and 2 of non-activated and 24h or 48h activated T cells cultured with Sema3A-CM or NSPI-CM. Results were obtained from 6 healthy donors. Kruskal-Wallis test. P value *<0.01, **<0.001, ***< 0.0001, ns- not significant

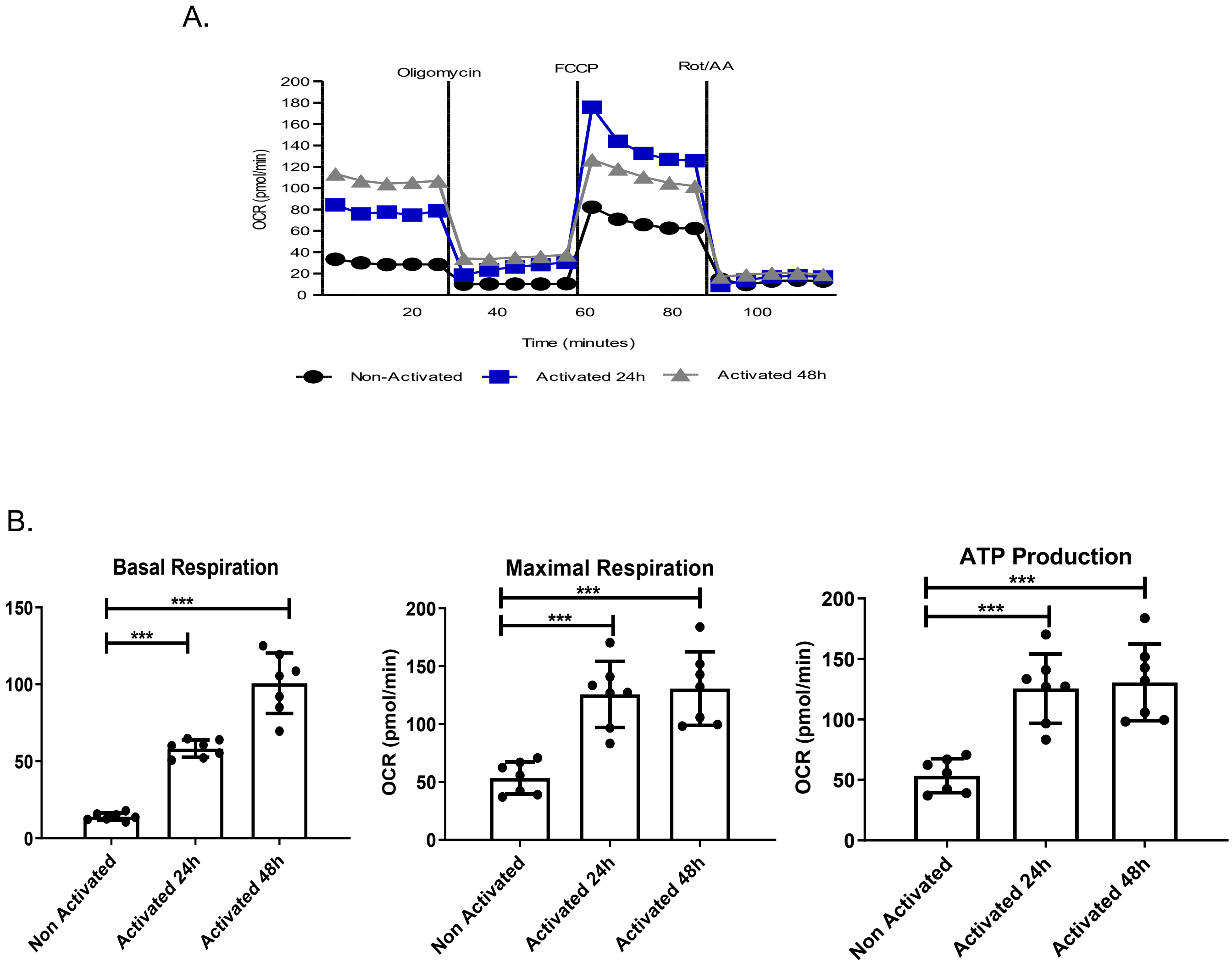

Supplemental Figure S2: 24h activation is sufficient to detect metabolic changes. A. Demonstrates a representative OCR graph of unstimulated and 24h or 48h activated T cells. B. Shows the measurement of three parameters: basal and maximal respiration as well as ATP production. Results were obtained from 7 healthy donors. Kruskal-Wallis test. P value ***<0.0005.

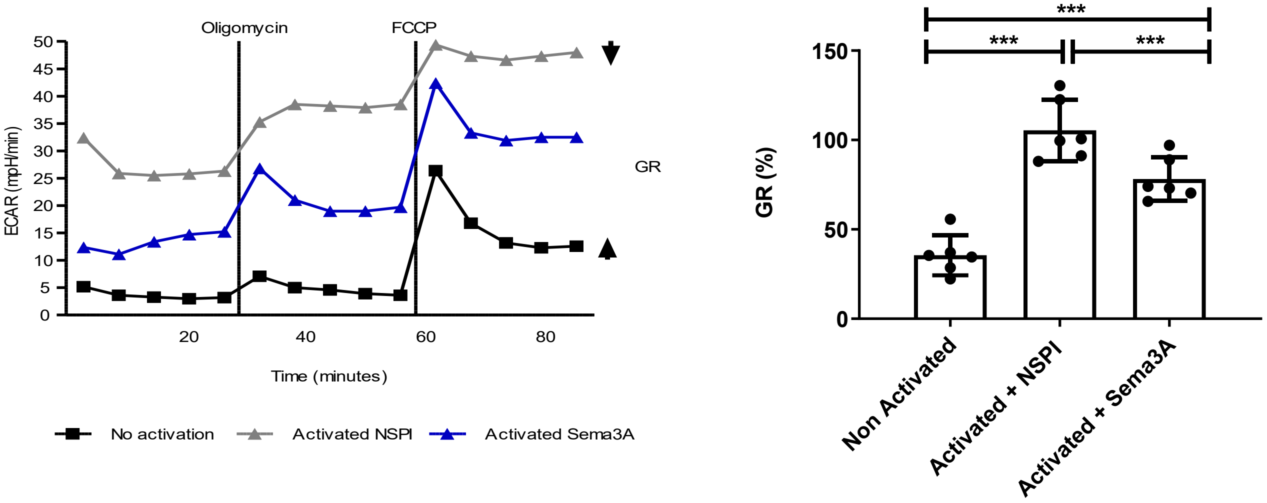

Supplemental Figure S3: GR is decreased in activated T cells treated with Sema3A. ECAR at baseline and after sequential treatment with oligomycin and FCCP. Kruskal-Wallis test. P value ***<0.0005

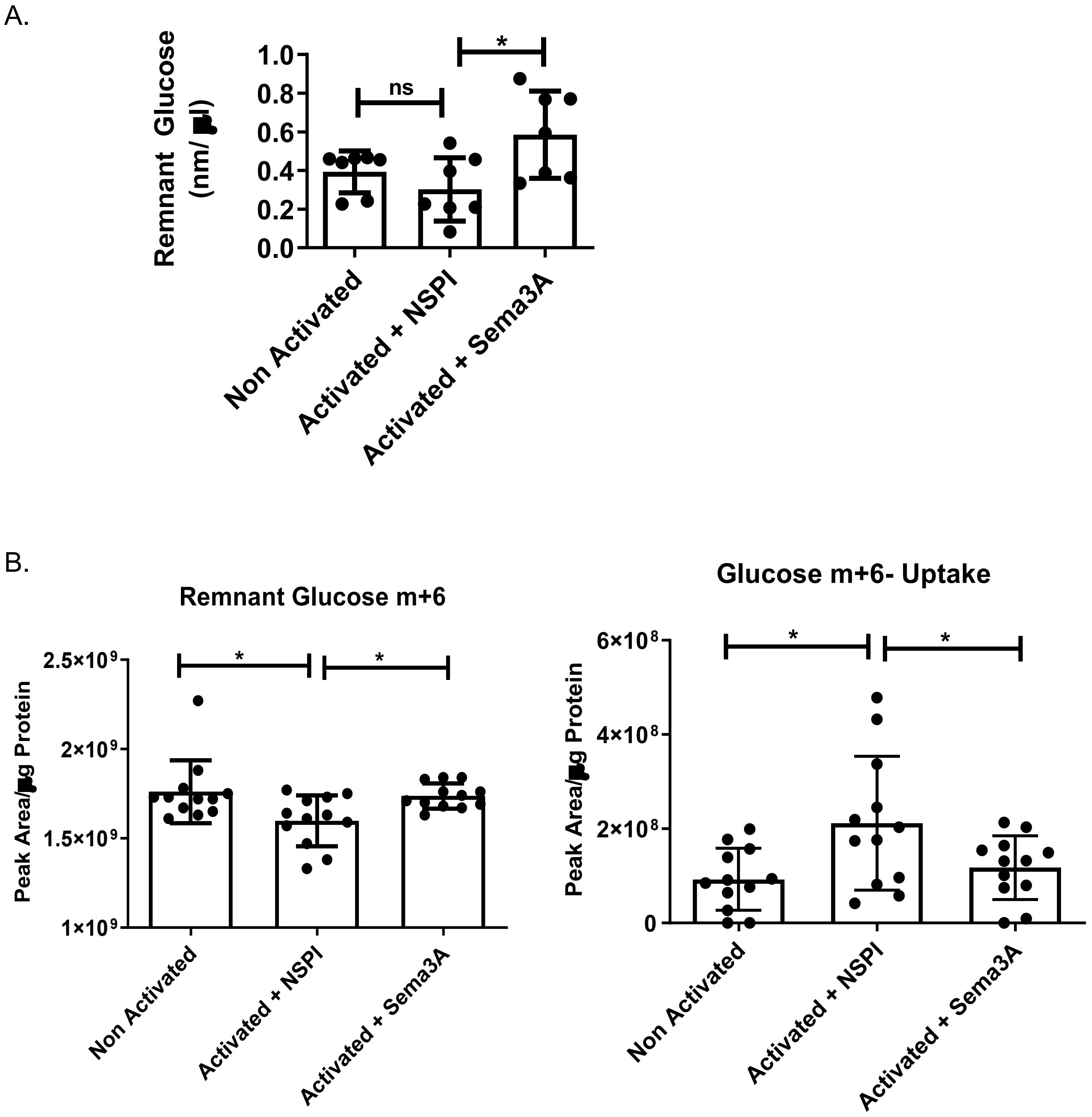

Supplemental Figure S4: Sema3A decreases glucose consumption in activated T cells. A. Glucose consumption Assay, shows the remnant glucose level in the cell media after a 24h activation with or without Sema3A. Results were obtained from at least 7 samples per treatment. B. Labeled glucose in cell media, analyzed using LC-MS. Remnant glucose and glucose uptake was calculated from 12 healthy donors. Kruskal-Wallis test. P value *<0.05, ns- not significant

A.

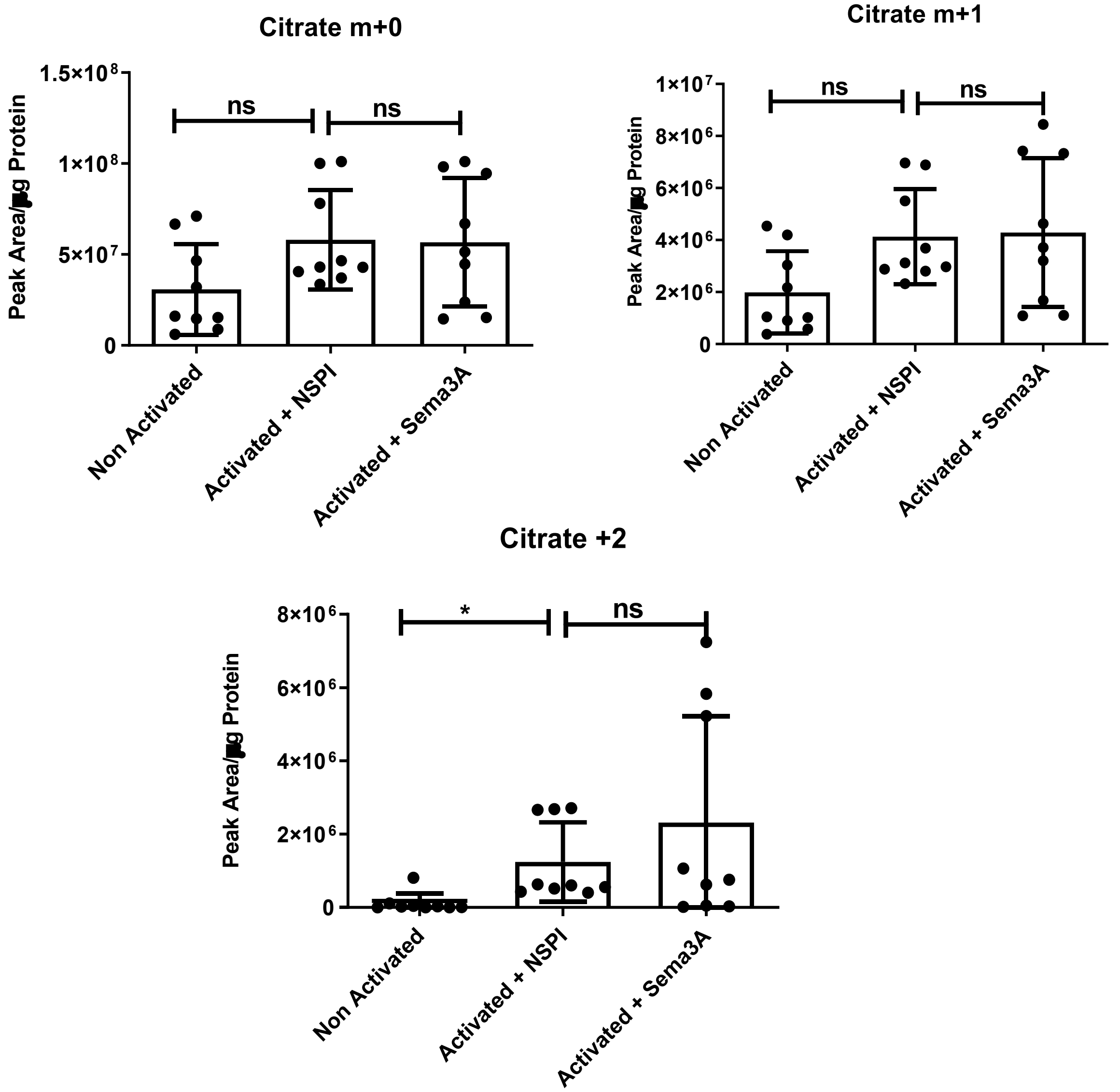
Citrate m+0
Peak Area/■g Protein
1.5×10^8
1×10^8
5×10^7
0
ns
ns
Non Activated
Activated + NSPI
Activated + Sema3A
Citrate m+1
Peak Area/■g Protein
1×10^7
8×10^6
6×10^6
4×10^6
2×10^6
0
ns
ns
Non Activated
Activated + NSPI
Activated + Sema3A
Citrate +2
Peak Area/■g Protein
8×10^6
6×10^6
4×10^6
2×10^6
0
*
ns
Non Activated
Activated + NSPI
Activated + Sema3A

B.

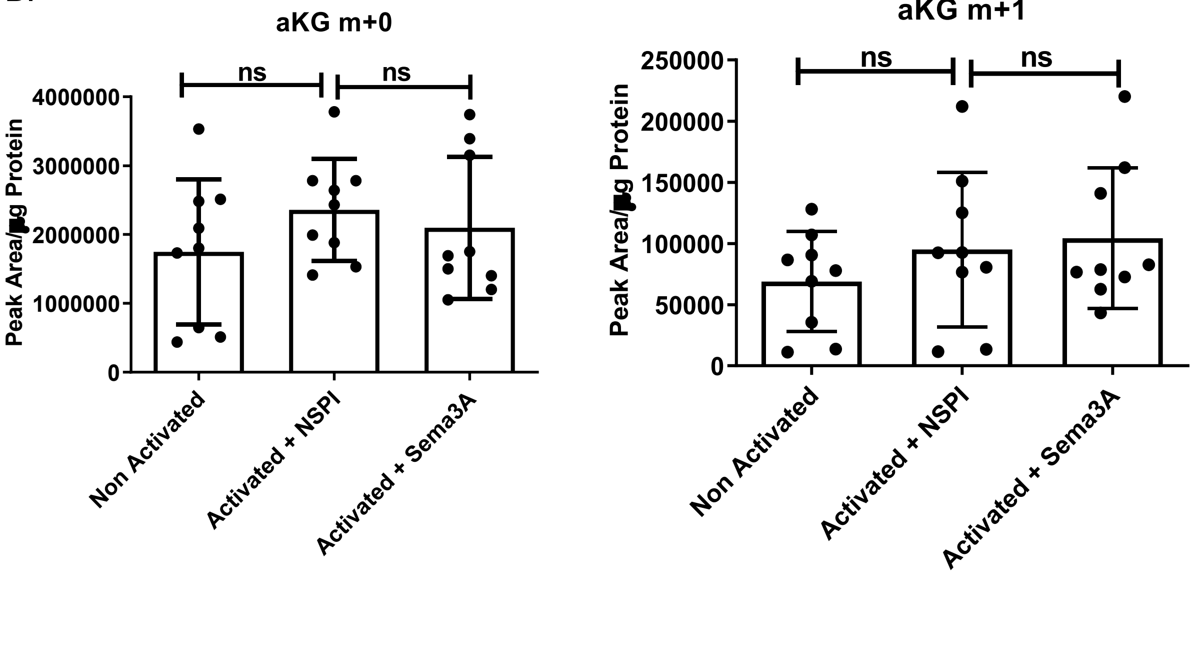
aKG m+0
Peak Area/■g Protein
4000000
3000000
2000000
1000000
0
ns
ns
Non Activated
Activated + NSPI
Activated + Sema3A
aKG m+1
Peak Area/■g Protein
250000
200000
150000
100000
50000
0
ns
ns
Non Activated
Activated + NSPI
Activated + Sema3A

C.

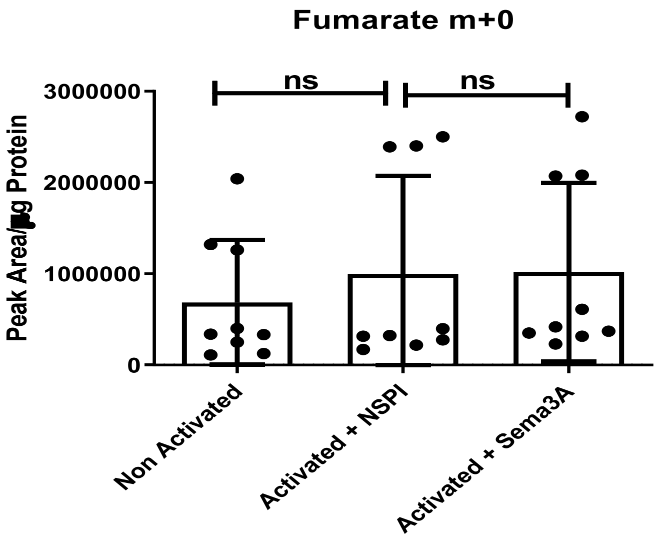

Supplemental Figure S5: Sema3A does not affect TCA metabolites. A-C. TCA cycle metabolites. Kruskal-Wallis test. P value *<0.05, ns- not significant
